# Induced Synthesis of Mycolactone Restores the Pathogenesis of *Mycobacterium ulcerans In Vitro* and *In Vivo*

**DOI:** 10.3389/fimmu.2022.750643

**Published:** 2022-03-24

**Authors:** Emily Strong, Bryan Hart, Jia Wang, Maria Gonzalez Orozco, Sunhee Lee

**Affiliations:** ^1^ Department of Microbiology and Immunology, University of Texas Medical Branch, Galveston, TX, United States; ^2^ Human Vaccine Institute, Duke University, Durham, NC, United States; ^3^ Department of Molecular Genetics and Microbiology, Duke University, Durham, NC, United States

**Keywords:** Buruli ulcer, mycobacteria, macrophages, mycolactone, host–microbe interaction, cytotoxicity, apoptosis, necrosis

## Abstract

*Mycobacterium ulcerans* is the causative agent of Buruli ulcer (BU), the third most common mycobacterial infection. Virulent *M. ulcerans* secretes mycolactone, a polyketide toxin. Most observations of *M. ulcerans* infection are described as an extracellular milieu in the form of a necrotic ulcer. While some evidence exists of an intracellular life cycle for *M. ulcerans* during infection, the exact role that mycolactone plays in this process is poorly understood. Many previous studies have relied upon the addition of purified mycolactone to cell-culture systems to study its role in *M. ulcerans* pathogenesis and host-response modulation. However, this sterile system drastically simplifies the *M. ulcerans* infection model and assumes that mycolactone is the only relevant virulence factor expressed by *M. ulcerans*. Here we show that the addition of purified mycolactone to macrophages during *M. ulcerans* infection overcomes the bacterial activation of the mechanistic target of rapamycin (mTOR) signaling pathway that plays a substantial role in regulating different cellular processes, including autophagy and apoptosis. To further study the role of mycolactone during *M. ulcerans* infection, we have developed an inducible mycolactone expression system. Utilizing the mycolactone-deficient *Mul*::Tn118 strain that contains a transposon insertion in the putative beta-ketoacyl transferase (*mup045*), we have successfully restored mycolactone production by expressing *mup045* in a tetracycline-inducible vector system, which overcomes *in-vitro* growth defects associated with constitutive complementation. The inducible mycolactone-expressing bacteria resulted in the establishment of infection in a murine footpad model of BU similar to that observed during the infection with wild-type *M. ulcerans*. This mycolactone inducible system will allow for further analysis of the roles and functions of mycolactone during *M. ulcerans* infection.

## Introduction

Mycobacteria are intracellular pathogens responsible for several diseases of global burden and concern. Tuberculosis (Tb) and leprosy (Hansen’s disease) infections, caused by *Mycobacterium tuberculosis* and *M. leprae*, respectively, are the two most common mycobacterial infections, with Buruli ulcer (BU) being the third most common disease presentation (1–4). BU is caused by *M. ulcerans* (*Mul*) and presents as a necrotizing cutaneous skin disease (5–7). Initially presenting as a papule, BU slowly progresses to a necrotic ulcer with extensive tissue loss, but it is typically painless due to the *Mul* expression of mycolactone (8, 9). The synthesis of mycolactone sets *Mul* aside from other mycobacteria (5). The biochemical machinery required for mycolactone synthesis by *Mul* is encoded for on the acquired megaplasmid (pMUM001) (10). Mycolactone has been readily identified in BU lesions and sera in humans and small animal models (11–15). Mycolactone concentrations in these lesions can be variable but range from 10 ng to 2 µg (13, 14, 16). Upon treatment and reduction of viable bacilli, mycolactone accumulation in BU lesions decreases; however, it remains detectable (13, 16).

One of the hallmarks of a BU lesion is its painless nature due to mycolactone signals through type 2 angiotensin II receptors and subsequent potassium-dependent neuron hyperpolarization (9, 17). It is well established that *in vitro*, mycolactone also causes cell rounding, cytoskeletal rearrangement, and detachment, which is caused by the interaction of mycolactone and Wiskott-Aldrich syndrome protein (WASP), leading to uncontrolled activation of ARP2/3. The unregulated ARP2/3 subsequently results in defective cell adhesions and directional migration (18). Additionally, it is cytotoxic and immunosuppressive, inhibiting the production of cytokines, chemokines, and adhesion molecules. The cellular effects of mycolactone can be attributed to its inhibitory effect on the Sec61 translocon (19–21). This interaction promotes apoptotic cell death *via* endoplasmic reticulum stress responses mediated by Bim (22–24), although Bim-induced apoptosis can be mediated by mTORC2 and Akt inhibition. The importance of Bim-dependent apoptosis during pathogenesis was demonstrated by *Mul* infection of Bim knockout mice, which did not develop necrotic BU lesions and were able to contain *Mul* multiplication (24).

Many of these mechanisms associated with mycolactone have been studied using synthetic mycolactone. Mycolactone alone has been linked to the induction of apoptosis *via* the inhibition of the mammalian target of rapamycin (mTOR) (24). However, it has been previously demonstrated that purified mycolactone cannot overcome the LPS activation of mTOR (25). Additionally, we have previously shown that mycobacteria are potent mTOR activators (26), which leads us to hypothesize that mycobacterial mTOR activation would not be overcome by mycolactone during *Mul* infection, and more complex regulation of apoptotic cell death by mycolactone may occur. For example, the histone methyltransferase SETD1B has also been identified as a novel mediator of mycolactone-induced cell death (27).

Some studies have been undertaken to demonstrate an intracellular infection stage for *Mul* very early during infection. These studies have highlighted the influx of neutrophils in response to early *Mul* infection in mice (6, 28, 29). Oliveira et al. (29) have proposed a model by which neutrophils and macrophages are recruited to the site of *Mul* infection. As the infection progresses, these cells become apoptotic and subsequently necrotic. This necrotic cell death facilitates bacterial escape from host phagocytes and the observation of the establishment of the acellular necrotic lesion characteristic of *Mul* infection (29, 30). The ability of *Mul* to persist past the intracellular phase, thereby establishing an ulcerative infection, has been linked to mycolactone. Mycolactone-competent bacteria induce necrosis both *in vivo* and *in vitro* leading to bacterial escape and establishing of the acellular necrotic lesion (29–31). Since many of these studies with extracted or purified mycolactone have been conducted using a sterile culture system, it raises the question of whether mycolactone can modulate the same host cell pathways in the presence of whole bacteria.

In the current study, we focused on determining the mechanism by which *Mul* controls its escape from macrophages. We found that mycolactone-competent *Mul* induces necrosis, enabling bacterial escape in primary bone marrow-derived macrophages and the human THP-1 monocyte-derived macrophages. Like most other pathogenic mycobacteria, *Mul* induces mTOR activation and limited autophagy. This finding is unlike observations of the role of synthetic mycolactone in host modulation; the addition of synthetic mycolactone inhibited mTOR activation and significantly induced autophagy, even during *Mul* infection, highlighting the limitations of using the synthetic mycolactone as a model to study *Mul*–mycolactone–host interactions. To overcome this discrepancy, we developed an inducible mycolactone expression system in *Mul*. Mycolactone induction in macrophages resulted in necrosis and bacterial escape, similar to wild-type *Mul*. In a mouse model of BU, the inducible mycolactone system resulted in progressive infection observed in wild-type *Mul* infection. This inducible system will help assess and examine *Mul*–mycolactone–host interactions, especially during the early stages of infection.

## Materials and Methods

### Bacterial Strains and Culture Conditions

All *Mul* strains were cultured at 32°C with shaking in Middlebrook 7H9 supplemented with 10% OADC (oleic acid, albumin, dextrose, catalase), 0.5% glycerol, and 0.02% tyloxapol or in Middlebrook 7H10 supplemented with 10% OADC and 0.5% glycerol with or without antibiotics as per requirements (hygromycin, 50 μg/ml or kanamycin, 25 μg/ml). *Escherichia coli* strains used for cloning were grown on LB agar or broth with or without antibiotics as per requirements (kanamycin 50 μg/ml or hygromycin, 100 μg/ml).

### Antibodies and Other Reagents

Antibodies were purchased from Cell Signaling Technology (Danvers, MA, USA) unless indicated otherwise and cataloged in **Table S1**. All reagents and media purchased from Sigma-Aldrich (St. Louis, MO, USA) unless otherwise stated.

### Macrophage Assays

Human THP-1 monocytes were maintained in supplemented Roswell Park Memorial Institute (RPMI)-1640 medium [bicarbonate buffered RPMI-1640 containing glutamine supplemented with 1% non-essential amino acids, 10% heat-inactivated fetal bovine serum (Corning, NY, USA), 1% HEPES, 1% sodium pyruvate, and 50 μM β-mercaptoethanol (RPMIc)] at 37°C with 5% CO_2_. THP-1 cells were seeded in 12-well plates at 5 × 10^5^ cells/well 72 h before infection or treatment. Monocytes were derived to macrophages by adding 10 ng/ml phorbol myristate acetate (PMA) for 48 h. Adhered derived macrophages were washed once in RPMIc and rested overnight in RPMIc before infection.

Bone marrow-derived primary cells were derived according to previously published methods (32). Briefly, marrow was flushed from tibias and femurs of 6- to 8-week-old C57BL/6J mice aseptically and cultured in non-tissue-culture-treated serological plates in RPMIc supplemented with 100 U/ml penicillin and 100 μg/ml streptomycin (RPMIcAbx). For macrophage differentiation, cells were seeded in 100-mm plates at 2 × 10^5^ cells/ml and differentiated by adding 15% L929 fibroblast-conditioned media for 6 days, followed by feeding with fresh media every 2 days. On day 6, adherent cells were washed with ice-cold phosphate-buffered saline (PBS) and detached by incubation on ice for 20 to 30 min in ice-cold PBS. BMDMs were seeded in 12-well plates at 5 × 10^5^ cells/well and allowed to adhere overnight in DMEMc.

Mycobacteria were grown to an optical density (OD_600_) of 0.6 to 0.8. After washing bacteria two times in PBS, bacteria were resuspended in RPMIc or DMEMc and de-clumped by centrifugation at 800 ×*g* for 8 min. De-clumped bacteria were used to infect macrophages at a multiplicity of infection (MOI) 10 unless otherwise stated. Infection was carried out for 4 h at 32°C, after which macrophages were washed 3 times with PBS and treated with 50 μg/ml gentamicin in complete media for 1 h to kill extracellular bacteria. Macrophages were rewashed 3 times with PBS, and assays were conducted in RPMIc or DMEMc for indicated times at 32°C with 5% CO_2_.

At indicated time points, culture supernatants were collected for LDH assay and bacterial enumeration. Cells were then harvested in radioimmunoprecipitation assay (RIPA) buffer (150 mM NaCl, 1% NP-40 or Triton X-100, 0.5% sodium deoxycholate, 0.1% SDS, 50 mM Tris–HCl, pH 8.0, 20 mM Tris–HCl, pH 7.5) for plating for intracellular survival or immunoblot analysis. For CFU enumeration, lysates were serially diluted and plated on 7H10 with appropriate antibiotics. Alternatively, macrophages were detached by incubation in ice-cold PBS containing 5 mM EDTA for 5 min on ice for staining.

### LDH Assay

At 72 h postinfection, cell-culture supernatants were collected for LDH analysis. The BioLegend LDH-Cytox Assay Kit was used, per the manufacturer’s instructions. Briefly, 50 µl of culture supernatants was added to 50 µl PBS in a 96-well plate. 100 µl of the working solution was added to wells and incubated at room temperature for 30 min. A 50-µl stop solution was added, and each well and absorbance were read to 490 nm. Cytotoxicity was calculated by subtracting the absorbance of untreated cells normalized to the absorbance of 100% lysed cells.

### Apoptosis Assay

Apoptosis was determined from collected cells using the GFP-certified Apoptosis/Necrosis detection kit (Enzo Life Sciences, Farmingdale, NY, USA) or CellEvent Caspase3/7 Green Detection Reagent (Invitrogen, Carlsbad, CA, USA), per manufacturers’ directions. For Apoptosis/Necrosis detection, cells were washed once in ice-cold PBS, resuspended in dual detection reagent (Annexin-V and 7-AAD), and incubated for 15 min. Cells were washed once more and resuspended in 2% PFA. For Caspase 3/7 activity, collected cells were washed once in ice-cold PBS. Macrophages were resuspended in detection reagent and incubated on ice for 30 min. Cells were washed once more in PBS and resuspended in 2% PFA. All samples were acquired on an Accuri C6 Plus Flow Cytometer (BD, Franklin Lakes, NJ, USA), and data were analyzed using FlowJo (Ashland, OR, USA).

### Immunoblotting

Macrophage cellular protein was prepared in 1× RIPA buffer, and bacterial cells were lysed by bead-beating with 1-mM silica zirconium beads in 0.05 M potassium phosphate and 0.02% β-mercaptoethanol. The protein concentration was determined by bicinchoninic acid (BCA) assay (Pierce). Aliquots of lysates containing 1 to 10 μg of protein were resolved on 12% SDS-PAGE gels at 180 V for 40 min. Proteins were transferred to 0.2 μm polyvinylidene difluoride (PVDF) using a Bio-Rad Trans-Blot Turbo at 2.5 A and 25 V for 5 to 10 min depending on molecular weight. PVDF membranes were blocked in 5% non-fat dry milk in 1× Tris-buffered saline (TBS) plus 0.1% Tween 20 (TBST) or OneBlock Western-CL Blocking Buffer (Genesee Scientific, San Diego, CA, USA) for LC3B blots at room temperature for at least 1 h. Primary antibodies at 1:5,000 dilution were incubated overnight at 4°C in TBST. The anti-Rabbit IgG-horseradish peroxidase (HRP) antibody (1:10,000) was added to membranes for 45 min in TBST. Proteins of interest were revealed using Clarity ECL (Bio-Rad) according to the manufacturer’s instructions. Blots were imaged using GE Amersham Imager 600, and densitometric analysis was conducted by ImageJ software (https://imagej.nih.gov/ij/links.html). The protein of interest was normalized to β-actin or GAPDH loading control to calculate autophagy levels (33).

### Cloning

The pTetR-*mup045* plasmid was constructed by cloning the PCR product (1,021 bp) of the *mup045* gene with a C-terminal HA tag into the backbone of the Tet-based expression vector pTACT13 (Addgene # 24784, Watertown, MA, USA), which was a gift from Tanya Parish. The *mup045*F (CCATGGGTGATTTGGAATGACATCTACATAAGTGG) and *mup045*HA (TTTAAACTAGGCGTAGTCCGGCACGTCGTACGGGTACGAAGTGGAGTGTCCGGGC) primers were used to amplify *mup045*. Both insert and vector were digested with NcoI and DraI. Mup045 expression was induced by adding 1 mg/ml anhydrotetracycline (Tet-ON).

### Mycolactone Extraction

Mycolactone was extracted from bacterial cultures as previously described (34). Briefly, a Folch extraction was done with 0.2 volumes of bacterial culture added to 0.8 volumes of 2:1 chloroform:methanol. This extraction was incubated for 2 h at room temperature with rocking. The solvent phase of the extraction was collected and dried, which was resuspended in ice-cold acetone and incubated overnight at -20°C. The acetone-insoluble precipitate was pelleted at 2,000 *×g* for 5 min, and acetone-soluble lipids were collected. These acetone soluble lipids are enriched for mycolactone. This fraction was again dried and resuspended in ethyl acetate for cytopathic assay.

### MTT Assay

L929 fibroblasts were seeded in 96-well plates at 2.5 × 10^5^ cells/ml in DMEMc and allowed to adhere overnight. Cells were treated with serial dilutions of synthetic mycolactone or extracted bacterial mycolactone for 72 h at 37°C. Synthetic mycolactone A/B were supplied by Dr. Yoshito Kishi (Harvard University), as ethanol-diluted solutions (1 mg/ml). The purity of synthetic mycolactone A/B was confirmed by 1H- and 13C-nuclear magnetic resonance and also by high-performance liquid chromatography. MTT [3-(4,5-dimethylthiazol-2-yl)-2,5-diphenyltetrazolium bromide] assay was carried out as per the manufacturer’s instructions (Abcam, Cambridge, MA, USA). Briefly, culture supernatant was removed from cells, replaced with serum-free media containing MTT reagent, and incubated at 37°C for 3 h. MTT solvent was then added and incubated at room temperature for 15 min. Absorbance was read at 595 nm using a microplate reader (Bio-Rad, Hercules, CA, USA).

### *In Vivo* Infection

All animal studies were approved by the institutional animal care and use committee of the University of Texas Medical Branch. Female C57BL/6J mice were obtained from The Jackson Laboratory between 6 and 8 weeks of age. Mice were infected with 1 × 10^4^ CFU subcutaneously in the left hind footpad. Footpad heights were measured fortnightly for 12 weeks. At weeks 4 and 10 postinfection, mice were euthanatized, and infected footpads were collected. Footpads were disinfected by soaking for 5 min in 70% ethanol followed by washing three times in PBS. Footpads were divided medially. For histology, half of the footpad was added to 10% neutral buffered formalin. The remaining footpad was diced into 2 ml PBS and homogenized using a probe homogenizer. One volume of disinfectant solution (4% NaOH, 2.9% sodium citrate, and 1% N-acetyl-L-cysteine) was added to the homogenate and incubated at room temperature for 20 min. A further 5 ml PBS was added to homogenates, and bacteria pelleted at 2,500 × g for 10 min. Pellets were resuspended in 1 ml PBS and serially diluted for CFU enumeration by plating.

### Statistical Analysis

GraphPad Prism 8 was used for all analyses. ANOVA was used to determine significance with Dunnett correction for multiple comparisons unless otherwise stated. A p-value of < 0.05 was considered to be significant.

## Results

### Mycolactone Induces Necrosis and Bacterial Egress During *M. ulcerans* Infection of Murine Macrophages

**Figure 1:**
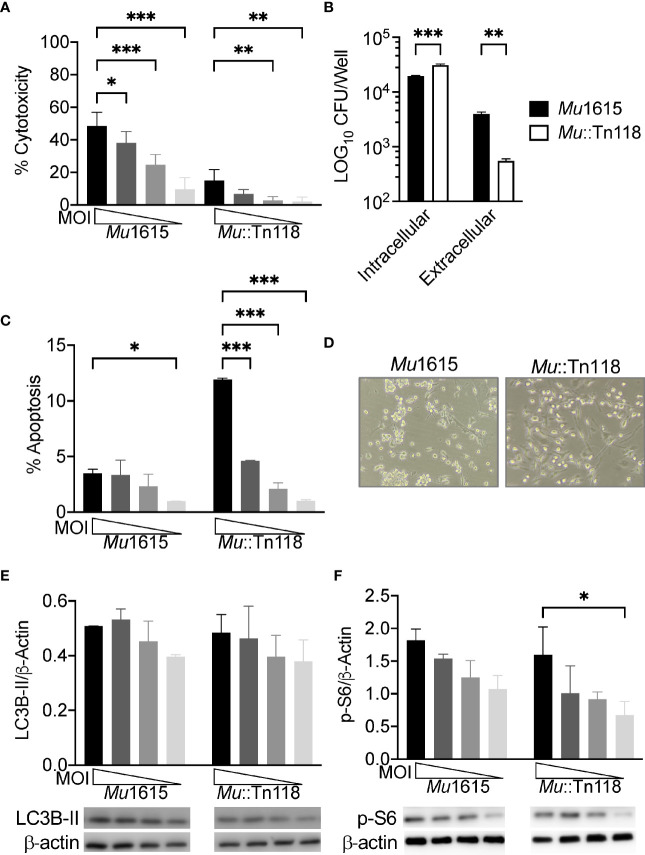
Mycolactone competent *M. ulcerans* induces necrosis, allowing for bacterial egress while maintaining mTOR activation during macrophage infection. **(A)** BMDMs infected with *Mu*1615 or *Mu*::Tn118 at MOI 10, 5, 2, and 1 were measured for LDH release at 72 h postinfection. **(B)** BMDM culture supernatant and cell lysate were plated for CFU enumeration at 72 h postinfection with *Mu*1615 and *Mu*::Tn118 at MOI 10. **(C)** Apoptosis was assayed by staining with Caspase 3/7 detection reagent in BMDMs infected with *Mu*1615 or *Mu*::Tn118 at MOI 10, 5, 2, and 1 at 72 h postinfection. **(D)** Representative bright-field microscopy image of BMDMs infected with *Mu*1615 or *Mu*::Tn118 at MOI 10, 72 h postinfection. Immunoblots were assayed from lysates of BMDMs infected with *Mu*1615 or *Mu*::Tn118 at MOI 10, 5, 2, and 1, 72 h postinfection for LC3B-II **(E)** or p-S6 **(F)**. Summary densitometric analysis was calculated by LC3B-II or p-S6 normalized to β-actin. The representative immunoblot is shown below densiometric analysis. All graphs represent one of two independent experiments with data expressed as mean ± SD. Significance was calculated by two-way ANOVA corrected by Dunnett’s test for multiple comparisons. *p ≤ 0.05, **p ≤ 0.01, ***≤ p 0.001.

An intracellular growth phase for *Mul* has been previously eluded to; however, mycolactone has also previously been described as inhibiting phagocytosis in macrophages (7, 30, 31). To study the exact role of mycolactone during an intracellular growth phase, we used the previously described mycolactone-deficient strain containing a transposon insertion in *mup045* (*Mu*::Tn118) and its parental strain (*Mu*1615) (7). Primary bone marrow-derived macrophages (BMDMs) from C57Bl/6J mice were infected with *Mu*1615 or *Mu*::Tn118 at a multiplicity of infection (MOI) of 10, 5, 2, and 1. At 72 h postinfection, cytotoxicity (LDH release) was measured in the macrophage culture supernatant (**Figure 1A**). Macrophages infected with *Mul* competent in mycolactone production (*Mu*1615) released increased LDH compared to macrophages infected with mycolactone-deficient *Mu*::Tn118 in a dose-dependent manner. While similar intracellular bacterial numbers were observed in *Mu*1615 and *Mu*::Tn118 infection 4 h postinfection (data not shown), significantly lower numbers of intracellular *Mu*1615 were observed at 72 h postinfection compared to *Mu*::Tn118 (**Figure 1B**). However, the number of *Mu*1615 was higher than *Mu*::Tn118 in the culture supernatant of these macrophage cultures. *Mu*1615 can egress from BMDMs more efficiently than *Mu*::Tn118 deficient in mycolactone synthesis. Thus, these data demonstrate that intracellular growth of *Mul*-producing mycolactone occurs by multiplication in individual macrophages followed by their lysis, egress of replicated bacilli.

Conversely, *Mu*::Tn118 induces significantly more apoptosis in BMDMs than the mycolactone-competent *Mu*1615 strain (**Figure 1C**). These results likely indicate that mycolactone plays a role in necrotic cell death, promoting bacterial egress from macrophages. Bright-field microscopy also shows this significantly increased lytic cell death in *Mu*1615-infected macrophages (**Figure 1D**). BMDMs infected with *Mu*1615 are more rounded and are less confluent than BMDMs infected with *Mu*::Tn118.

Synthetic and purified mycolactone have been previously shown to inhibit mTOR and Akt, resulting in BH3-only BCL-2-interacting mediator of cell death (Bim) protein activation and apoptosis (24). To determine if this translates to live *Mul* infection, we examined LC3B-II accumulation as a measure of autophagy and phospho-S6 (Ser235/236) as a measure of mTOR activation. We observed low levels of autophagy (**Figure 1E**) in *Mul*-infected BMDMs, like observations from other virulent mycobacteria-infected macrophages (26). As with these other well-studied mycobacterial infections, we observed significant levels of mTOR activation (**Figure 1F**). Autophagy and mTOR activation were increased in a dose-dependent manner. There was no difference in autophagy or mTOR activation between the mycolactone-competent and -deficient strains, indicating that bacterial mycolactone cannot overcome the mTOR activation by *Mul*.

### Mycolactone Induces Necrosis and Bacterial Egress During *M. ulcerans* Infection of Human Monocyte-Derived Macrophages

**Figure 2:**
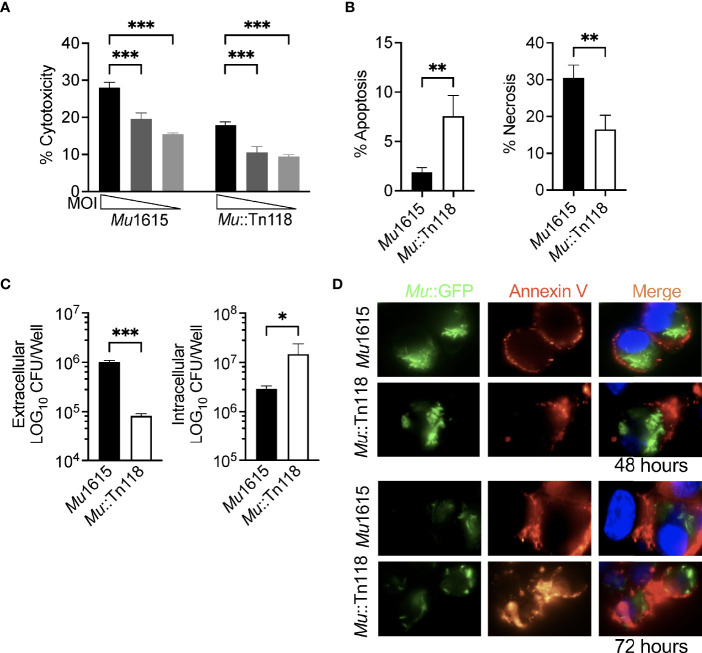
Mycolactone competent *Mul* induces necrosis leading to bacterial escape. **(A)** THP-1 monocyte-derived macrophages infected with *Mu*1615 or *Mu*::Tn118 at MOI 10, 5, and 2 were measured for LDH release at 72 h postinfection. **(B)** Apoptosis and necrosis were assayed by staining with Annexin-V and 7-AAD in THP-1 monocyte-derived macrophages infected with *Mu*1615 or *Mu*::Tn118 at MOI 10, 72 h postinfection. **(C)** THP-1 monocyte-derived macrophage culture supernatant and lysate were plated for CFU enumeration at 72 h postinfection with *Mu*1615 and *Mu*::Tn118 at MOI 10. **(D)** THP-1 monocyte-derived macrophages were infected with *Mu*1615::GFP or *Mu*::Tn118::GFP at MOI 10. At 48 and 72 h, postinfection cells were stained with Annexin-V and visualized by microscopy. Representative images are shown from two independent experiments. Green, Mu::GFP; red, Annexin V; and Blue, DAPI. All graphs represent one of two independent experiments with data expressed as mean ± SD. Significance was calculated by two-way ANOVA corrected by Bonferroni test for multiple comparisons **(A)** or T-test **(B, C)**. *p ≤ 0.05, **p ≤ 0.01, ***≤ p 0.001.

Many studies have demonstrated a role for mycolactone in apoptosis induction during BU infection (8, 22–24, 35). While this seems likely, we hypothesized that mycolactone also induced necrosis to facilitate *Mul*’s escape from phagocytic cells during the early stage of infection. To facilitate the study of the bacterial egress, we determined if THP-1 human monocyte-derived macrophages behave similarly to primary murine macrophages. Monocyte-derived THP-1 macrophages were infected with *Mul* at an MOI of 10, 5, and 2. At 72 h postinfection, the cytotoxicity from these infected macrophages was determined (**Figure 2A**). A similar dose response in cytotoxicity to the infected BMDMs was observed, and we subsequently chose to do all the following experiments in this human monocytic cell line. As with the BMDMs, we once again demonstrated reduced cytotoxicity in *Mu*::Tn118-infected macrophages compared to *Mu*1615-infected cells. To differentiate between apoptosis and necrosis, we stained infected macrophages with Annexin-V and 7-AAD. Cells positive for only Annexin-V were determined to be apoptotic (**Figure 2B**), while cells positive for both Annexin-V and 7-AAD were determined to be necrotic, which thus have a permeable membrane allowing for bacterial egress from the macrophage. Synthetic and purified mycolactones have been previously shown to inhibit mTOR and Akt, resulting in BH3-only BCL-2-interacting mediator of cell death (Bim) protein activation and apoptosis (24). In line with results from BMDMs, these data support the proposed hypothesis that mycolactone-induced lytic cell death led to bacterial escape, which was further evidenced by the increased extracellular bacteria in the macrophage culture supernatant 72 h postinfection (**Figure 2C**).

We further demonstrated this decreased intracellular CFU by microscopically examining THP-1 monocyte-derived macrophages infected with *Mu*1615::GFP or *Mu*118::GFP at an MOI of 10, 48, and 72 h postinfection (**Figure 2D** and **Supplementary Figure 1**). These cells were stained with Annexin-V to mark apoptosis and necrosis. Annexin-V staining was observed in both *Mu*1615- and *Mu*118-infected cells. However, a reduced number of intracellular bacteria were observed at 72 h postinfection compared to 48 h postinfection in *Mu*1615::GFP-infected cells than *Mu*118::GFP-infected cells, demonstrating *Mu*1615::GFP escape due to the increased necrosis during mycolactone competent infections (**Supplementary Figure 1**).

### Synthetic Mycolactone Induces Apoptosis, Necrosis, and Autophagy During *M. ulcerans* Infection

**Figure 3:**
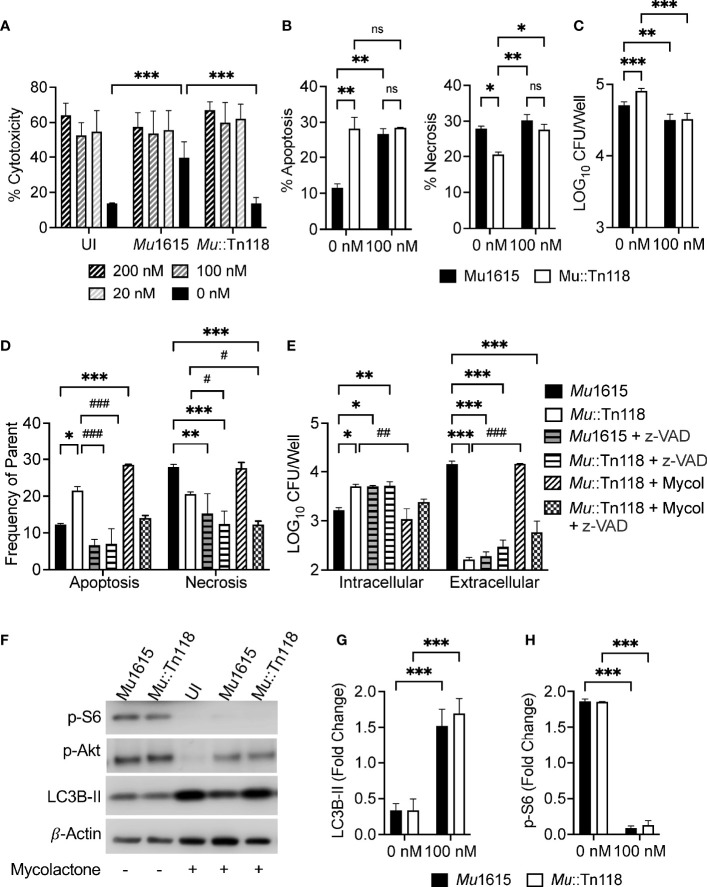
Synthetic mycolactone induces apoptosis, necrosis, and autophagy during *Mul* infection. **(A)** THP-1 monocyte-derived macrophages infected with *Mu*1615 or *Mu*::Tn118 at MOI 10 were treated with 200, 100, 20, and 0 nM of synthetic mycolactone. At 72 h postinfection, LDH release was measured. **(B)** Apoptosis and necrosis were assayed by staining with Annexin-V and 7-AAD in THP-1 monocyte-derived macrophages infected with *Mu*1615 or *Mu*::Tn118 at MOI 10 ± 100 nM mycolactone, 72 h postinfection. **(C)** THP-1 monocyte-derived macrophage cell lysates were plated for CFU enumeration at 72 h postinfection with *Mu*1615 and *Mu*::Tn118 at MOI 10 ± 100 nM mycolactone treatment. **(D)** THP-1 monocyte-derived macrophages infected with *Mu*1615 or *Mu*::Tn118 at MOI 10 were treated with pan-caspase inhibitor, Z-VAD-FMK (z-VAD), or 100 nM synthetic mycolactone (Mycol). At 72 h postinfection, apoptosis and necrosis were assayed by staining with Annexin-V and 7-AAD. * and # indicate the comparison with *Mu*1615 or *Mu*::Tn118, respectively. **(E)** THP-1 monocyte-derived macrophage culture supernatant and cell lysate were plated for CFU enumeration at 72 h postinfection with *Mu*1615 and *Mu*::Tn118 at MOI 10 with and without treatment of z-VAD or 100 nM Mycolactone. **(F)** Immunoblots were assayed from lysates of THP-1 monocyte-derived macrophages infected with *Mu*1615 or *Mu*::Tn118 at MOI 10 ± 100 nM mycolactone treatment, 72 h post-infection for LC3B-II, p-Akt, or p-S6. **(G, H)** Summary densitometric analysis was calculated by LC3B-II or p-S6 normalized to β-actin. All graphs represent one of two independent experiments with data expressed as mean ± SD. Significance was calculated by two-way ANOVA corrected by Dunnett’s test for multiple comparisons. ns, not significant. *p ≤ 0.05, **p ≤ 0.01, ***≤ p 0.001, ^#^p ≤ 0.05, ^##^p ≤ 0.01, ^###^≤ p 0.001.

During infection with mycolactone-competent *Mul*, we observed a significant activation of mTOR. However, it is documented that synthetic mycolactone inhibits signaling of mTORC1/2, resulting in dephosphorylation and inactivation of the Akt kinase and induction of Bim-dependent apoptosis *via* the mTORC2–Akt–FoxO3 axis (24). The Akt-targeted transcription factor, FoxO3, is the central transcriptional regulator of Bim-induced apoptosis in mycolactone-treated cells. To confirm that synthetic mycolactone can modulate this pathway during *Mul* infection similar to bacterial mycolactone modulation, we treated infected THP-1 monocyte-derived macrophages with increasing concentrations of synthetic mycolactone. Synthetic mycolactone induced high levels of cytotoxicity at all concentrations in both uninfected and infected macrophages, as expected (**Figure 3A**). These data indicated that synthetic mycolactone induces lytic cell death, as observed in mycolactone-competent *Mul* infections.

As all tested concentrations of mycolactone induced cytotoxicity and many other studies have used 80 to 200 nM of synthetic mycolactone, we chose to conduct the remaining studies with 100 nM of mycolactone. Unlike *Mu*1615 infection, the addition of synthetic mycolactone induced apoptosis and necrosis with mycolactone-competent and -deficient *Mul* infection (**Figure 3B**), suggesting that synthetic mycolactone may modulate apoptosis induction differently to mycolactone produced during *Mul* infection. As expected, we observed that intracellular bacteria are decreased in macrophages 72 h postinfection during mycolactone treatment (**Figure 3C**). These data, taken together, highlight a critical role of mycolactone in modulating host death pathways.

To confirm that necrosis induced by mycolactone was responsible for bacterial egress from macrophages, we treated infected THP-1-derived macrophages with the pan-caspase inhibitor Z-VAD-FMK. Pan caspase inhibition demonstrated reduced levels of apoptosis and necrosis during *Mul* infection or synthetic mycolactone treatment (**Figure 3D**). In macrophages where caspase-dependent necrosis was inhibited, we also observed increased intracellular bacteria and decreased extracellular bacteria (**Figure 3E**).

Indicative of the shortcomings of the synthetic mycolactone model to determine its role during the early stages of infection, we found significant differences in autophagy induction and mTOR modulation by synthetic mycolactone and bacterial mycolactone. The addition of synthetic mycolactone to *Mul*-infected macrophages resulted in increased autophagy as measured by LC3B-II accumulation (**Figures 3F, G**). As predicted, this is due to synthetic mycolactone’s ability to inhibit mTOR, as measured by p-S6 and p-Akt (Ser473) accumulation, during infection (**Figures 3F, H**). mTOR activation and autophagy activation were simultaneously increased in a dose-dependent manner and responded to the presence of synthetic mycolactone (**Supplementary Figure 2**). These data indicated that while synthetic mycolactone is a valuable resource to help understand its ability to modulate host systems, it may overly simplify the complex infection dynamic to represent reality closely. To study the mycolactone–*Mul*–host infection dynamics more accurately, we have developed an inducible expression system for mycolactone.

### Development of an Inducible Mycolactone Expression System

**Figure 4:**
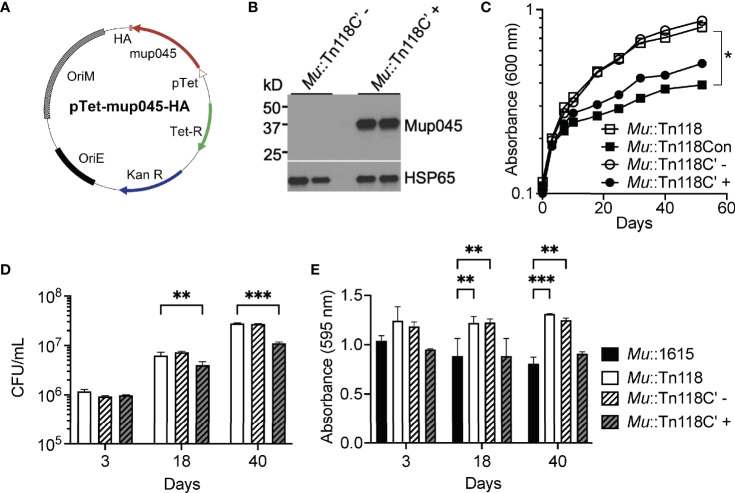
Expression of the mycolactone accessory gene (*mup045*) inhibits bacterial growth and restores mycolactone production in *Mu*::Tn118. **(A)** Plasmid map for pTetR-*mup045* is shown. Expression of *mup045* (blue) C-terminally tagged with HA is driven by the tetracycline promoter (pink). **(B)** Confirmation of expression of HA-tagged *mup045* in *Mu*::Tn118 under the tetracycline-inducible promoter (*Mu*::Tn118C′) with (+) and without (-) 1 μg/ml anhydrous tetracycline (aTCN) at day 25 growth in 7H9. **(C)** Growth curve of recombinant *Mu*::Tn118 in 7H9 constitutively expressing *mup045* (*Mu*::Tn118Con) or expressing *mup045* under the aTCN-inducible promoter (*Mu*::Tn118C′) ± 1 μg/ml aTCN. **(D)** Correlating CFU from growth curves at days 3, 18, and 40 of growth in 7H9. **(E)** Cytotoxicity of mycolactone extracted from 7H9 cultures at days 3, 18, and 40 as determined by MTT assay in L929 cells. All graphs represent one of three independent experiments with data expressed as mean ± SD. Significance was calculated by two-way ANOVA corrected by Dunnett’s test for multiple comparisons. *p ≤ 0.05, **p ≤ 0.01, ***≤ p 0.001.

To comprehensively study the role of mycolactone during *Mul* infection, we developed an inducible *mup045* expression system to restore mycolactone synthesis in the *Mu*::Tn118 mutant strain with a transposon insertion in *mup045*. Utilizing a previously described tetracycline-inducible promoter (36), the *mup045* gene from *Mu*1615 was cloned into the tetracycline-inducible vector with a 3′ hemagglutinin (HA) tag (pTetR-mup045, **Figure 4A**) and transformed into *Mu*::Tn118, resulting in *Mu*::Tn118C′. The *mup045*-HA expression was confirmed by Western blot (**Figure 4B**), induced by anhydrous tetracycline (aTCN). The presence of the tetracycline-inducible construct (*Mu*::Tn118C′) did not affect the growth of *Mu*::Tn118 *in vitro* (**Figure 4C**). However, the induced expression of *mup045*-HA by aTCN addition (*Mu*::Tn118C′+) or the constitutive expression of *mup045* in *Mu*::Tn118 (*Mu*::Tn118Con) decreased *in vitro* growth. A significant decrease in bacterial CFU from those liquid cultures at 18 and 40 days was shown (**Figure 4D**). A constitutive expression of *mup045* under the HSP60 promotor is highly toxic *in vitro* (data not shown), underscoring an advantage of the inducible expression system described here. To confirm that *mup045*-HA expression resulted in mycolactone synthesis, we extracted mycolactone by Folch’s extraction method at days 3, 18, and 40 of *in vitro* growth. The significantly increased cytotoxicity of extractions from *Mu*1615 and *Mu::Tn118C’* growth with aTCN to L929 fibroblasts was observed by MTT assay compared to *Mu*::Tn118 or the uninduced *Mu*::Tn118C′ (**Figure 4E**). Apoptosis and necrosis induction by the inducible mycolactone producers were also determined on L929 fibroblasts (**Supplementary Figure 3**). The synthetic mycolactone and mycolactone produced during infection cause substantial necrosis and limited apoptosis in fibroblast. These results suggest that mycolactone is an essential substance for the induction of necrosis, and there is a difference in the activity of mycolactone on different cell types.

### Induced Synthesis of Mycolactone Restores Necrosis, Allowing *M. ulcerans* to Escape From Infected Macrophages

**Figure 5:**
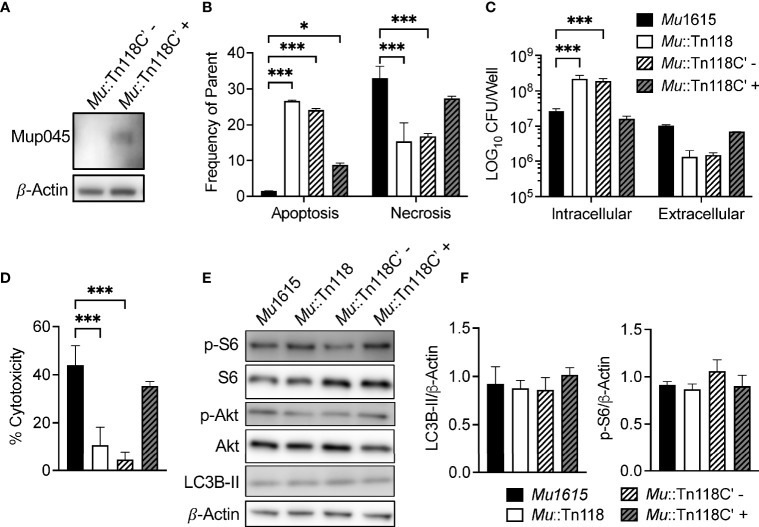
Induced synthesis of mycolactone restores *Mu*::Tn118 necrosis allowing for bacterial escape from macrophages. **(A)** Representative immunoblot for HA-tagged *mup045* from THP-1 monocyte-derived macrophages infected with *Mu::Tn118C’* ± 1 μg/ml aTCN at MOI 10, 72 h postinfection. A representative experiment from three independent experiments is shown. **(B)** Apoptosis and necrosis were assayed by staining with Annexin-V and 7-AAD in THP-1 monocyte-derived macrophages infected with *Mu*1615, *Mu*::Tn118 or *Mu::Tn118C’* ± 1 μg/ml aTCN at MOI 10, 72 h postinfection. **(C)** THP-1 monocyte-derived macrophage lysates and culture supernatants were plated for CFU enumeration at 72 h postinfection with *Mu*1615, *Mu*::Tn118 or *Mu::Tn118C’* ± 1 μg/ml aTCN at MOI 10. **(D)** THP-1 monocyte-derived macrophages infected with *Mu*1615, *Mu*::Tn118, or *Mu*::Tn118C′ at MOI 10, and culture supernatants were measured for LDH release at 72 h postinfection. **(E)** Immunoblots were assayed from lysates of THP-1 monocyte-derived macrophages infected with *Mu*1615, *Mu*::Tn118 or *Mu::Tn118C’* ± 1 μg/ml aTCN at MOI 10, 72 h postinfection for LC3B-II, p-Akt, total Akt, phosphor-S6, or total S6. **(F)** Summary densitometric analysis was calculated by LC3B-II or p-S6 normalized to β-actin. All graphs represent one of two independent experiments with data expressed as mean ± SD. Significance was calculated by two-way **(B, C, F)** or one-way **(D)** ANOVA corrected by Bonferroni or Dunnett’s test for multiple comparisons. *p ≤ 0.05, ***≤ p 0.001.

Because we developed this system to enable the study of the role of mycolactone during early *Mul* infection, we first confirmed that this inducible system behaves as *Mu*1615 does. *Mu*1615, *Mu*::Tn118, and *Mu*::Tn118C′ were used to infect the THP-1 cells at MOI 10 for 3 days. The addition of 1 µg/ml aTCN during the *Mu*::Tn118C′ infection successfully induced the expression of *mup045*-HA in this system (**Figure 5A**). *Mu*1615 and *Mu*::Tn118C′ (+ aTCN) induced high levels of necrosis, resulting in increased bacterial egress from THP-1 cells 72 h postinfection compared to *Mu*::Tn118 infection (**Figures 5B, D**). Conversely and as observed in **Figure 2**, *Mu*1615 and *Mu*::Tn118C′ (+ aTCN) induced low levels of apoptosis and reduced intracellular bacteria in THP-1 cells 72 h postinfection compared to *Mu*::Tn118 infection. As expected, the induction of *mup045*-HA expression resulted in increased cytotoxicity in THP-1 macrophages 72 h postinfection than *Mu*::Tn118 infection, behaving like *Mu*1615 infection (**Figure 5D**). We also observed that necrosis induction by mycolactone-producing strains is significantly higher than non-producing *Mul* strains at 48, 72, and 96 h postinfection of THP-1 cells (**Supplementary Figure 4**). The enhanced necrosis correlates with a higher extracellular bacterial number for *Mu*1615 and *Mu::Tn118C’* + strains (**Supplementary Figure 5**). Taken together, these data demonstrate that our tetracycline-inducible mycolactone synthesis system successfully complements *Mu*::Tn118 during macrophage infection. Perhaps even more significantly, we further demonstrated that phosphorylation and total abundance of S6 ribosomal and Akt proteins were not elevated, and autophagy inhibition is maintained in THP-1-derived macrophages infected with *Mu*::Tn118C′ (+aTCN), as observed in *Mu*1615 and *Mu*::Tn118 infection (**Figures 5E, F**, and **Supplementary Figure 6**).

### Induced Synthesis of Mycolactone Restores *Mu*::Tn118 Function in a Murine Model of BU

**Figure 6:**
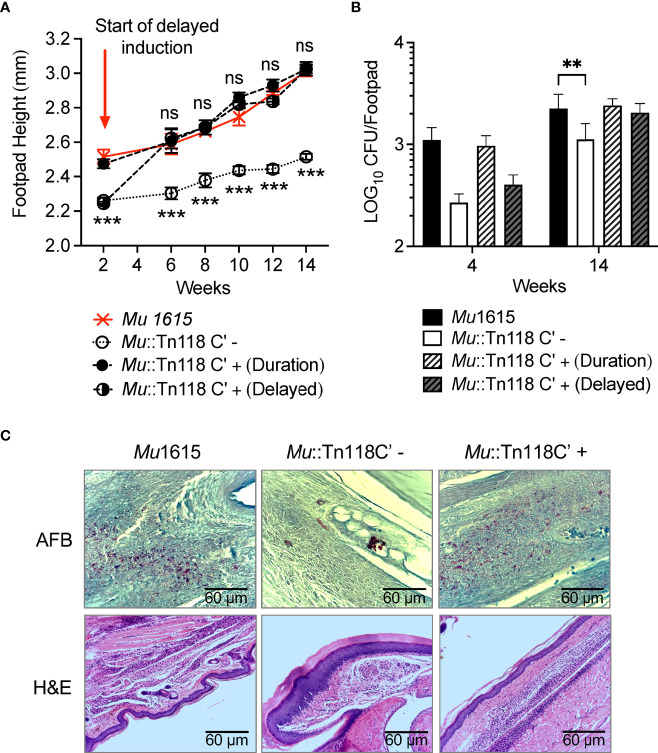
Induced synthesis of mycolactone restores *Mu*::Tn118 function in a murine model of BU. **(A)** Footpad heights of C57Bl/6J mice infected with 1 × 10^4^ bacteria of *Mu*1615 or *Mu::Tn118C’* ± doxycycline in left hind footpads. Mean ± SEM of two independent experiments (n = 8 mice). **(B)** Bacterial burden of infected left hind footpads of infected mice 4 and 14 weeks postinfection. Mean ± SD of two independent experiments (n = 8 mice). Significance was calculated by two-way ANOVA corrected by Dunnett’s test for multiple comparisons. NS, not significant. **p ≤ 0.01, ***≤ p 0.001. **(C)** Representative H&E- and AFB-stained footpad sections are shown for mice at 14 weeks postinfection.

Although an *in vitro* system to study mycolactone function during bacterial infection is a valuable tool, studying these interactions *in vivo* is vital to confirming the physiological relevance of such studies. To validate if the inducible system described above works *in vivo*, we utilized the mouse footpad model of BU infection. C57BL/6J mice were infected in the left hind footpad with 1 × 10^4^ CFU, and footpad swelling was monitored for 14 weeks. Mice were infected with *Mu*1615 and *Mu*::Tn118C′. *Mu::Tn118C’*-infected groups were left untreated or treated with 1 mg/ml doxycycline in drinking water for ad libitum administration for 14 weeks of the experimental period (duration), or treatment was started 2 weeks postinfection (delayed) and maintained for the following 12 weeks. Doxycycline treatment resulted in footpad swelling in infected mice like that observed during *Mu*1615 infection (**Figure 6A**). *Mu*1615 and *Mu*::Tn118C′ (+doxycycline)-infected mice showed increased bacterial persistence for 4 and 10 weeks postinfection than *Mu::Tn118C’*-infected mice that did not receive doxycycline treatment (**Figure 6B**). This result was further confirmed by the increased presence of acid-fast bacilli as observed by histology (**Figure 6C**). Hematoxylin and eosin (H&E) staining also revealed increased cellular infiltrate during infection with *Mu*1615 and *Mu*::Tn118C′ (+doxycycline) than seen with *Mu*::Tn118C′ (-doxycycline). As we utilized a relatively low-dose infection model, by 14 weeks postinfection, we did not observe the establishment of an extensive necrotic infection in the footpad.

Our data taken together demonstrate an essential role for mycolactone during *Mul* infection. Using a tetracycline-inducible mycolactone synthesis system, we have identified an important role for mycolactone in inducing necrosis during *Mul* infection. Mycolactone synthesis is essential for bacterial egress from macrophages and the establishment of necrotic lesions during infection. Bacterial synthesis of mycolactone is unable to overcome mTOR induction and autophagy inhibition by *Mul*, unlike previous observations that mycolactone is a potent mTOR inhibitor and autophagy activator. Thus, the tetracycline-inducible mycolactone synthesis system developed in this study will help assess the mechanisms by which mycolactone induces necrosis during *Mul* infection.

## Discussion

Virulent *Mul* secretes mycolactone, a cytotoxic exotoxin with a critical pathogenic role. *In vitro* assays have suggested that *Mul* uptake by macrophages is inhibited by mycolactone, and specimens from patients and mouse footpad lesions have shown a concentration of extracellular bacilli in central necrotic acellular areas. Thus, *Mul* has been previously classified as an extracellular pathogen. However, more recent *in vivo* data have demonstrated that upon *Mul* infection, there is likely a brief intracellular growth stage before establishing the necrotic ulcer (7, 30, 31). *In vivo*, intracellular growth of mycobacteria appears to occur by cycles of multiplication in individual macrophages followed by their lysis, egress of replicated bacilli, extracellular proliferation, and entry of these bacilli into new macrophages where the growth cycle is repeated (8, 24, 35). However, it is unclear if mycolactone is a prerequisite for the *Mul* life cycle.

We demonstrated that the mycolactone-competent strain *Mu*1615 is cytotoxic in THP-1-derived macrophages, primary murine BMDMs, and mouse fibroblast L929 cells. This toxicity was identified as necrosis (**Figures 1**, **2**), which seems responsible for the increased bacterial egress from macrophages. The bacterial egress induced by bacterial or synthetic mycolactone was inhibited by adding a pan-caspase inhibitor, further supporting that caspase-dependent necrosis is the route by which *Mul* escapes macrophages. Similarly, virulent *M. tuberculosis* inhibits apoptosis and, instead, induces necrosis. Necrosis leads to intercellular dissemination of *M. tuberculosis* (37). Torrado *et al.* demonstrated that mycolactone-producing *Mul* is efficiently phagocytosed by murine macrophages, indicating that the extracellular location of *Mul* is not a result of inhibition of phagocytosis (30). Instead, an essential role of necrosis for bacterial egress from macrophages during *Mul* infection was demonstrated in this study. Of note, others have previously shown distinct morphological features indicating necrosis during *Mul* infection both *in vivo* and from biopsies from BU patients (29, 38).

The observation that cytotoxicity associated with mycolactone produced by *Mul* infection is necrosis, and not apoptosis as others reported (8, 24, 35), can be explained. First, the necrosis we observed is in fact secondary necrosis, which happens when phagocytes do not successfully take up apoptotic cells, and apoptotic cells proceed to the phase of late apoptosis (39, 40). Second, most studies tested the cytotoxicity of mycolactone (either purified from culture supernatant or synthetic) on L929 cells, not macrophages. One study compared mycolactone’s effect on macrophages and fibroblasts and demonstrated that apoptosis occurred after 2 days of treatment with mycolactone isolated from culture supernatants in the macrophage cell line as opposed to after 3 days of treatment for the fibroblasts (8). Our studies demonstrated much less apoptosis induction with *Mu*::Tn118 on L929 cells than THP1 or BMDM cells. Third, infection with pathogenic or non-pathogenic *Mul* modifies the interaction with the host and induces cell death pathways, different from the responses by purified mycolactone (without infection). It will be important to evaluate and compare the molecular mechanisms of different cell deaths by synthetic mycolactone or purified mycolactone from culture supernatants or mycolactone produced during the infection of *Mul*.

Similarly, a recent study observed that only mycolactone-producing *Mul* or vehicles collected from the infected macrophages infected with such *Mul* could induce pyroptosis with phenotypes of the production of IL-1b, targeting NLRP3/1 inflammasomes (41). The study further supports our result that mycolactone-producing *Mu1615* prominently induces necrotic cell death. Both necrosis and pyroptosis represent inflammatory lytic cell death pathways and are reported to have the same physiological outcomes (membrane permeabilization and inflammatory cytokine release) (42). Our future studies will directly investigate if necrosis induced with an inducible mycolactone expression system is indeed pyroptotic or a regulated cell death that mimics features of apoptosis and necrosis, known as necroptosis (43–46).

Synthetic mycolactone can inhibit Akt by mTOR inhibition, driving apoptotic cell death (24); however, bacterially synthesized mycolactone cannot overcome mTOR or Akt activation by *M. ulcerans* infection, like the synthetic substitute (**Figure 3F**). While the addition of synthetic mycolactone did restore *Mu*::Tn118 necrosis, it also induced significantly more apoptosis than in WT *Mu*1615 infection (**Figures 3B, C**), unlike observations during mycolactone-competent bacterial infections. This result is not because 100 nM of synthetic mycolactone is significantly higher than mycolactone produced during infection since we observed the same findings with treatments of mycolactone as low as 1 nM (data not shown). Thus, data presented here suggest a more complex regulation than the direct inhibition of mTOR and Akt *via* mycolactone.

mTOR activation and autophagy induction simultaneously increased in a MOI-dependent manner (**Supplementary Figure 2**). The pathway for the mTOR-independent autophagy induction during mycobacterial infection is still unknown. Notably, we have previously demonstrated that all mycobacteria species tested are potent mTOR activators (26, 47). When synthetic mycolactone is added to the infected THP-1 cells, mTOR activation is significantly decreased to a similarly low level regardless of MOI (as indicated by the p-S6 signal), and autophagy increases, likely resulting from inhibition of the mTOR-dependent signaling. However, we still cannot explain the LC3BII level of uninfected cells treated with mycolactone being higher than the infected cells with mycolactone. The result may suggest that *Mul* encodes autophagy-inhibiting proteins seen in *M. tuberculosis* that interfere with autophagy activation (48–51). Although the mechanisms involved in the interactions between host and *Mul* are not known, these results again emphasize that studying the role of mycolactone in *Mul* pathogenesis and host response modulation should be interpreted with caution when experiments involve adding purified mycolactone to cell culture systems. Of significance, it has recently been shown that inhibition of Sec61 by mycolactone triggers an increase in initiation of canonical autophagy, which is translationally regulated through EIF2S1 phosphorylation (52).

Induction of mycolactone synthesis under its native promoter would be the most robust model for studying the role of mycolactone. It has recently been demonstrated that mycolactone synthesis genes, including *mup045*, are expressed by the SigA like promoter (53). Interestingly, it appears that *Mul* controls mycolactone synthesis posttranscriptionally in a mechanism that has not yet been studied and deciphered (53, 54). In a spontaneous healing murine model of BU, deficient levels of mycolactone are observed during the healing stage of the disease. Transcriptional analysis of the mycolactone synthesis genes during this time demonstrates no differences in mRNA levels compared to the ulcerative stage of infection. These observations indicate that *Mul* must adapt to its metabolic levels to survive spontaneous healing, thus lowering mycolactone production posttranscriptionally (55). If mycolactone levels *in vivo* are controlled by a posttranscriptional mechanism relating to bacteria metabolism, the induced expression of mycolactone under a tetracycline-constitutive promoter will provide valuable and accurate insights into the role of mycolactone during bacterial infection.

The mycolactone inducible system that we have created successfully complements *Mu*::Tn118 to WT function *in vitro* and *in vivo*. Induction of mycolactone synthesis successfully induces necrosis and bacterial egress with low levels of apoptosis in macrophages. Importantly, bacterial synthesis of mycolactone does not overcome the mTOR activation induced by *Mul* infection (**Figure 5**). It also restores bacterial function in a murine model of BU (**Figure 6**). Treatment of mice postinfection with doxycycline for the experiment duration demonstrated that footpad swelling increased bacterial survival, and increased cellular infiltrates as observed in WT *Mu*1615 infection compared to mice infected with *Mu*::Tn118C′ not treated with doxycycline (uninduced). Strikingly, mice infected with *Mu*::Tn118C′ without mycolactone synthesis induction display no signs of disease and very low bacterial numbers. This reduced bacterial survival and pathology of mycolactone-deficient bacteria can be rescued upon induction of mycolactone synthesis up to 2 weeks postinfection. The ability to induce mycolactone synthesis *in vivo* and *in vitro* during critical stages of infections will allow many further studies into the exact role of mycolactone during the intracellular infection stage and the progression of the necrotic disease.

Here, we chose to study the role of mycolactone in macrophages, a standard model for studying host–mycobacterial interactions. However, in both a guinea pig and a murine model of BU infection, neutrophils are the most common cell type identified cuffing the necrotic lesion (7, 28). Neutrophilic cellular debris was also identified within the necrotic lesion (28). These data suggest that *Mul* could reside in neutrophils upon host infection. However, mycolactone-deficient *Mul* is observed in granulomatous structures with a high proportion of macrophages in this guinea pig model of BU (28). In a peritoneal murine model of BU, a significant influx of both neutrophils and macrophages has been observed in response to several clinical isolates, demonstrating that macrophages are an essential immune infiltrate in response to *Mul* infection (29). As such, it will be interesting to examine the role of mycolactone in the induction of necrosis in neutrophils.

In summary, we have demonstrated that *Mul* drives necrosis to facilitate bacterial escape from phagocytic cells and establish the necrotic lesion associated with BU. We developed an inducible expression construct for mycolactone synthesis, restoring *Mu*::Tn118 to wild-type function *in vitro* and *in vivo*. Considering the discrepancy observed in *Mul* disease progression and pathogenesis with synthetic mycolactone, establishing these inducible strains will help further studies into how mycolactone modulates apoptosis and necrosis during the early stages of infection *in vivo*. We anticipate obtaining valuable insight into BU disease progression and another tool to develop more advanced treatment options.

## Data Availability Statement

The original contributions presented in the study are included in the article/**Supplementary Material**. Further inquiries can be directed to the corresponding author.

## Ethics Statement

The animal study was reviewed and approved by the UTMB IACUC Committee.

## Author Contributions

ES and SL conceived the study. ES designed and conducted experimental procedures. BH conducted molecular generation of the inducible expression system. JW performed Western analysis for manuscript revision. MG assisted with *in vivo* studies. ES and SL interpreted the data. SL supervised the study. ES wrote the manuscript, and SL provided the overall editing for the manuscript. All authors contributed to the manuscript revision, read and approved the submitted version.

## Funding

This research was supported by the National Institute of Allergy and Infectious Diseases (R01 AI127711). The funders had no role in the study design, data collection, interpretation, or decision to submit the work for publication. SL was supported by UTMB institutional funds.

## Conflict of Interest

The authors declare that the research was conducted without any commercial or financial relationships that could be construed as a potential conflict of interest.

## Publisher’s Note

All claims expressed in this article are solely those of the authors and do not necessarily represent those of their affiliated organizations, or those of the publisher, the editors and the reviewers. Any product that may be evaluated in this article, or claim that may be made by its manufacturer, is not guaranteed or endorsed by the publisher.

## Supplementary Material

Supplementary Figure 1 Representative images of DAPI, Annexin V, and M. ulceransfluorescence staining are shown. THP-1 monocyte-derived macrophages were infected withMu1615::GFP or Mu::Tn118::GFP at MOI 10. At 48- and 72-hours, post-infection cells werestained with Annexin-V and visualized by microscopy. Representative images are shown from oneof two independent experiments shown. Cell nucleus was visualized by a blue signal (DAPI), ared signal visualized annexin V, and M. ulcerans was visualized by a green signal.

Supplementary Figure 2 Immunoblots were assayed by Western blot for LC3B, p-Akt, and p-S6. THP-1 cells were infected with Mu1615 or Mu::Tn118 at MOI 2, 5, and 10 and incubated +/-80 ng/mL mycolactone for 72 hours. UI, uninfected.

Supplementary Figure 3 Cytotoxicity of mycolactone synthetized or produced during infectionof L929 fibroblast was determined. L929 was infected with the indicated strains at MOI 10 ortreated with 50 ng/ml of synthetic mycolactone. Apoptosis/Necrosis staining at day 3 and 5- postinfection/treatment was performed. All graphs represent one of three independent experiments with data expressed as mean ± SD. Significance was calculated by Two-Way ANOVA corrected by Dunnett's Test for multiple comparisons. * p ≤ 0.05, ** p ≤ 0.01, **** ≤ p 0.0001

Supplementary Figure 4 Flow cytometry analysis of apoptosis and necrosis over time is shown. A) Apoptosis and necrosis were assayed by staining with Annexin-V and 7-AAD in THP-1 monocyte-derived macrophages infected with Mu1615, Mu::Tn118 or Mu::Tn118C' ± 1 μg/mL aTCN at MOI 10, 48, 72, and 96 hours post-infection. B) THP-1 monocyte-derived macrophages infected with Mu1615 or Mu::Tn118 at MOI 10 were measured for LDH release at 72 and 96 hours post-infection. All graphs represent one of two independent experiments with data expressed as mean ± SD. Significance was calculated by Two-Way ANOVA corrected by Bonferroni Test for multiple comparisons. * p ≤ 0.05, ** p ≤ 0.01, *** ≤ p 0.001

Supplementary Figure 5 THP-1 monocyte-derived macrophage lysates (intracellular) and culture supernatants (extracellular) were plated for CFU enumeration at 1, 2, 3, and 4 days postinfection with Mu1615, Mu::Tn118, or Mu::Tn118C' ± 1 μg/mL aTCN at MOI 10. Day 0 indicates the number of intracellular bacilli at 4 hours post-infection and indicates the infected bacilli. All graphs represent one of two independent experiments with data expressed as mean ± SD.

Supplementary Figure 6 Immunoblots were assayed from lysates of THP-1 monocyte-derived macrophages infected with Mu1615 or Mu::Tn118 at MOI 10 ± 100 nM mycolactone treatment, 72 hours post-infection for total Akt or S6.
