# Supplementary figures and images for: Induced Synthesis of Mycolactone Restores the Pathogenesis of Mycobacterium ulcerans In Vitro and In Vivo
Source: Front Immunol. 2022 Mar 24;13:750643. doi: 10.3389/fimmu.2022.750643 (PMC8988146; doi:10.3389/fimmu.2022.750643)

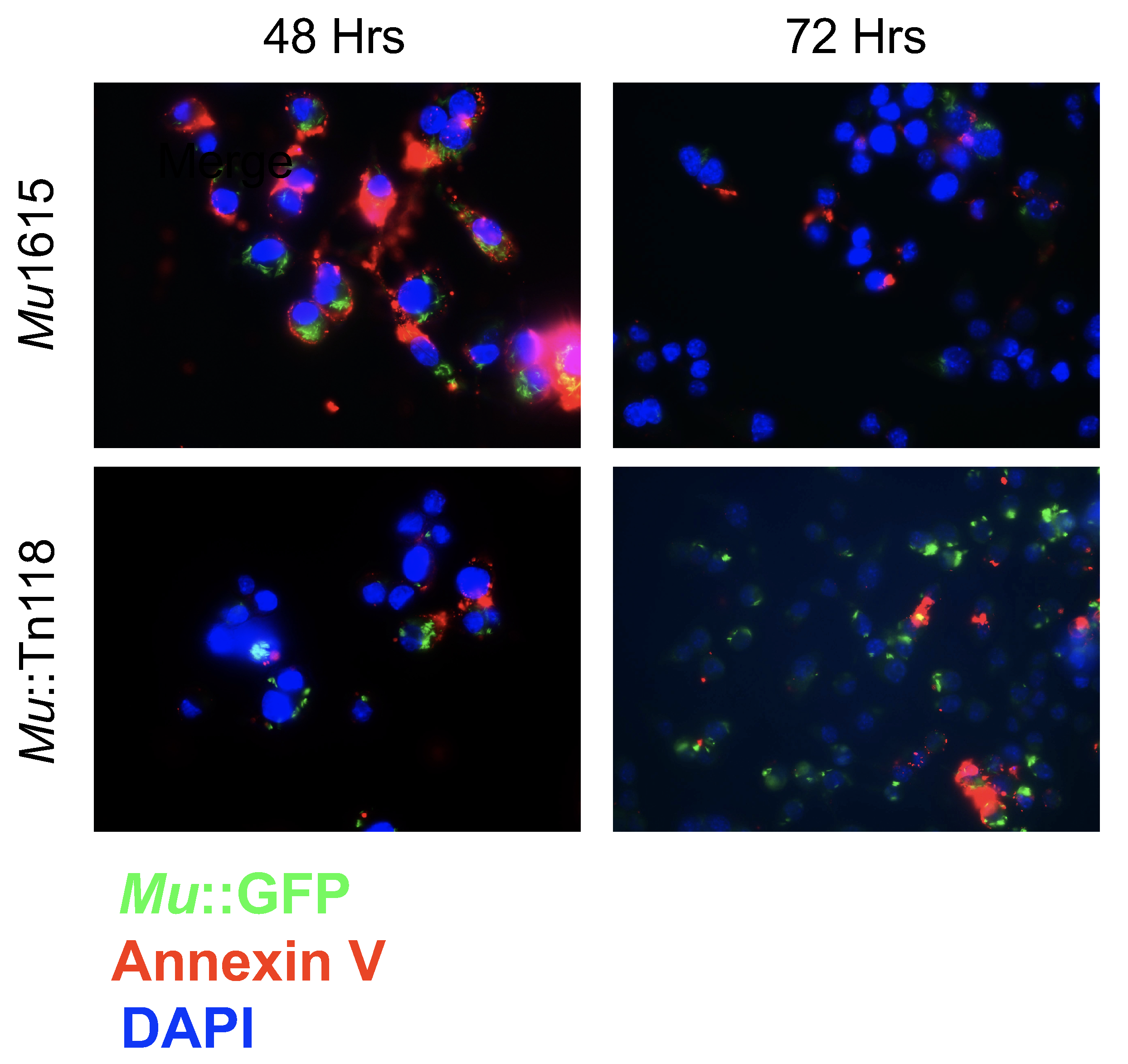

Supplement: Supplementary Figure 1 — Representative images of DAPI, Annexin V, and M. ulceransfluorescence staining are shown. THP-1 monocyte-derived macrophages were infected withMu1615::GFP or Mu::Tn118::GFP at MOI 10. At 48- and 72-hours, post-infection cells werestained with Annexin-V and visualized by microscopy. Representative images are shown from oneof two independent experiments shown. Cell nucleus was visualized by a blue signal (DAPI), ared signal visualized annexin V, and M. ulcerans was visualized by a green signal. [file Image_1.tiff]

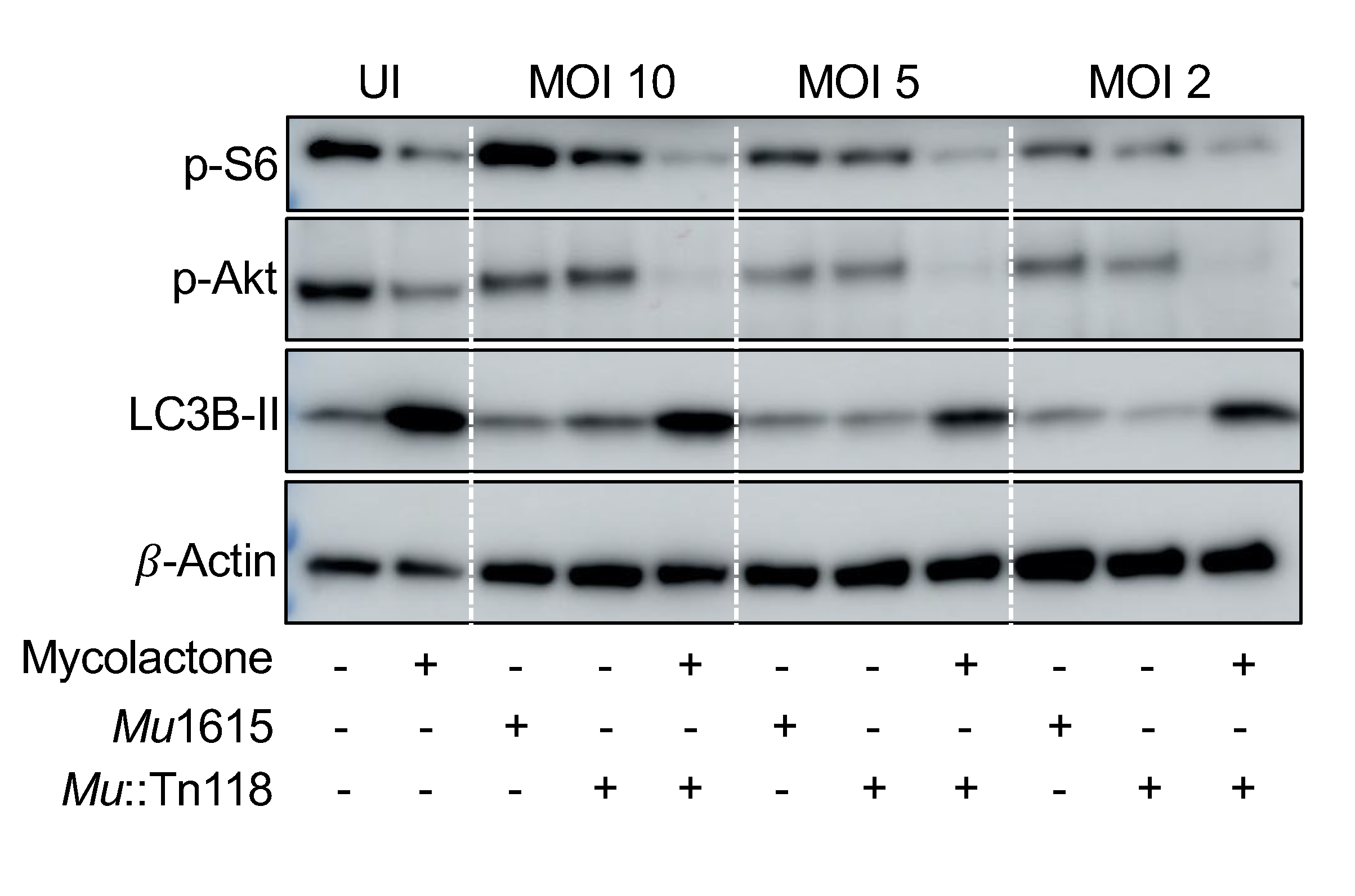

Supplement: Supplementary Figure 2 — Immunoblots were assayed by Western blot for LC3B, p-Akt, and p-S6. THP-1 cells were infected with Mu1615 or Mu::Tn118 at MOI 2, 5, and 10 and incubated +/-80 ng/mL mycolactone for 72 hours. UI, uninfected. [file Image_2.tiff]

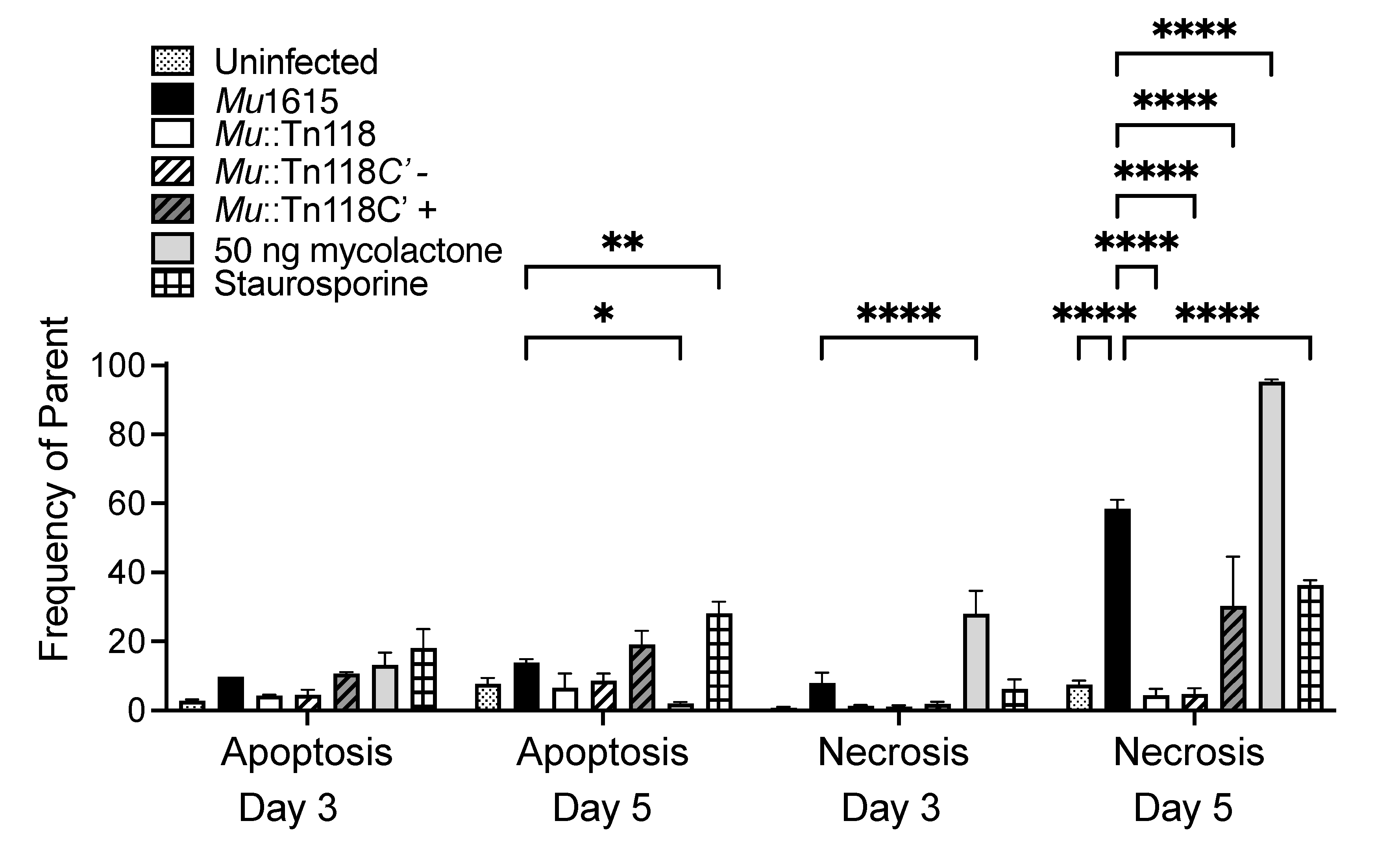

Supplement: Supplementary Figure 3 — Cytotoxicity of mycolactone synthetized or produced during infectionof L929 fibroblast was determined. L929 was infected with the indicated strains at MOI 10 ortreated with 50 ng/ml of synthetic mycolactone. Apoptosis/Necrosis staining at day 3 and 5- postinfection/treatment was performed. All graphs represent one of three independent experiments with data expressed as mean ± SD. Significance was calculated by Two-Way ANOVA corrected by Dunnett's Test for multiple comparisons. * p ≤ 0.05, ** p ≤ 0.01, **** ≤ p 0.0001 [file Image_3.tiff]

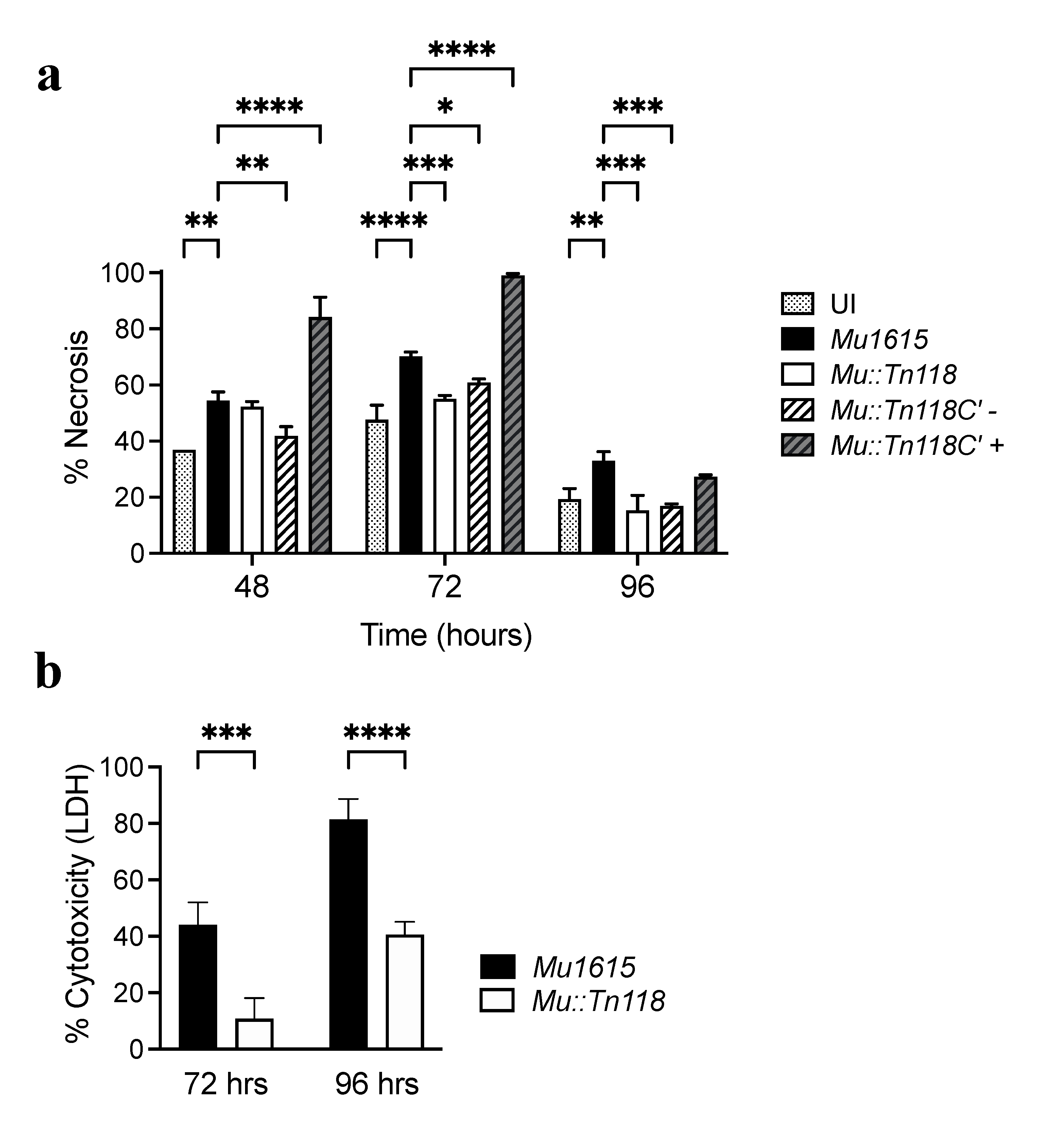

Supplement: Supplementary Figure 4 — Flow cytometry analysis of apoptosis and necrosis over time is shown. A) Apoptosis and necrosis were assayed by staining with Annexin-V and 7-AAD in THP-1 monocyte-derived macrophages infected with Mu1615, Mu::Tn118 or Mu::Tn118C' ± 1 μg/mL aTCN at MOI 10, 48, 72, and 96 hours post-infection. B) THP-1 monocyte-derived macrophages infected with Mu1615 or Mu::Tn118 at MOI 10 were measured for LDH release at 72 and 96 hours post-infection. All graphs represent one of two independent experiments with data expressed as mean ± SD. Significance was calculated by Two-Way ANOVA corrected by Bonferroni Test for multiple comparisons. * p ≤ 0.05, ** p ≤ 0.01, *** ≤ p 0.001 [file Image_4.tiff]

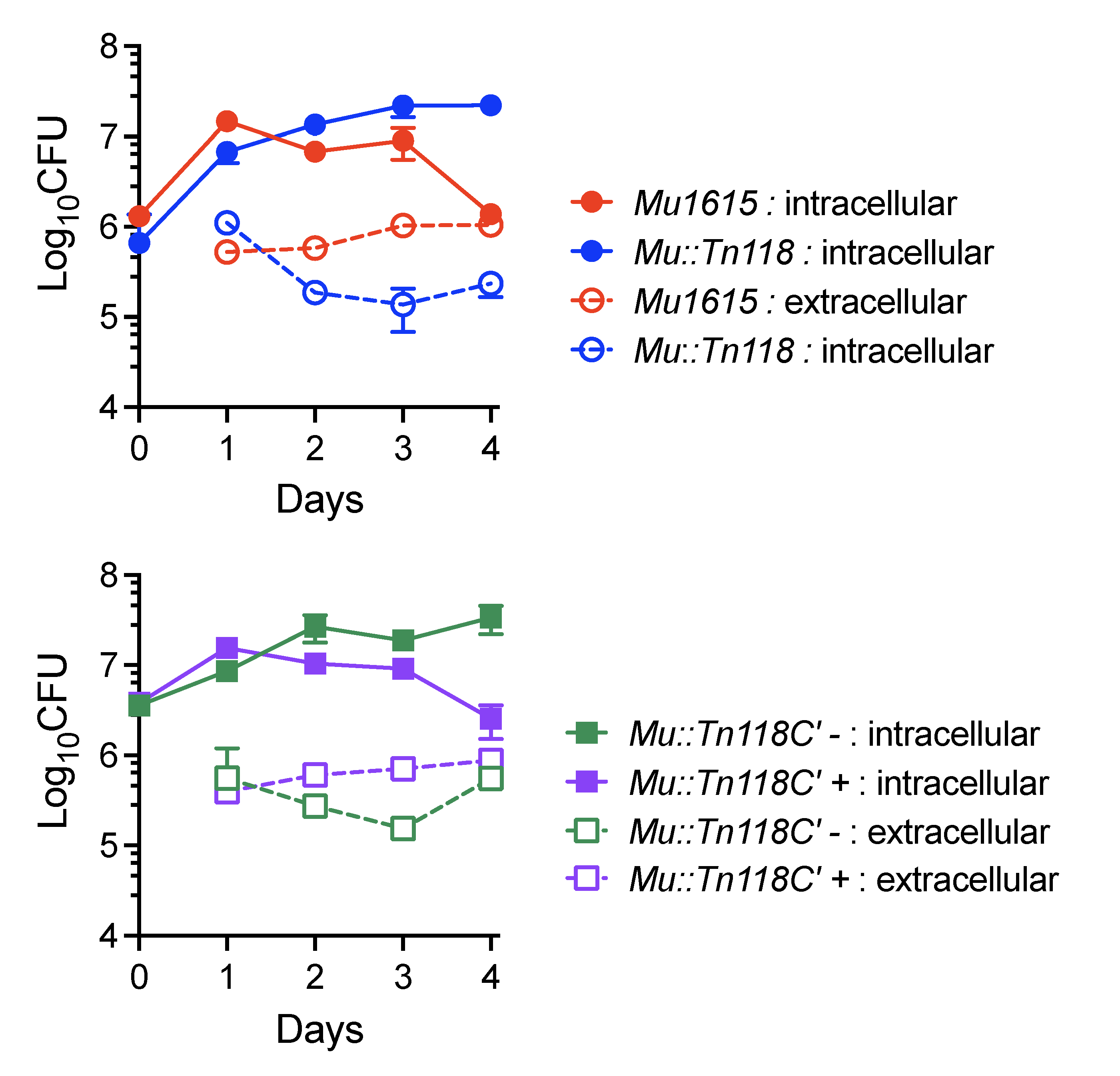

Supplement: Supplementary Figure 5 — THP-1 monocyte-derived macrophage lysates (intracellular) and culture supernatants (extracellular) were plated for CFU enumeration at 1, 2, 3, and 4 days postinfection with Mu1615, Mu::Tn118, or Mu::Tn118C' ± 1 μg/mL aTCN at MOI 10. Day 0 indicates the number of intracellular bacilli at 4 hours post-infection and indicates the infected bacilli. All graphs represent one of two independent experiments with data expressed as mean ± SD. [file Image_5.tiff]

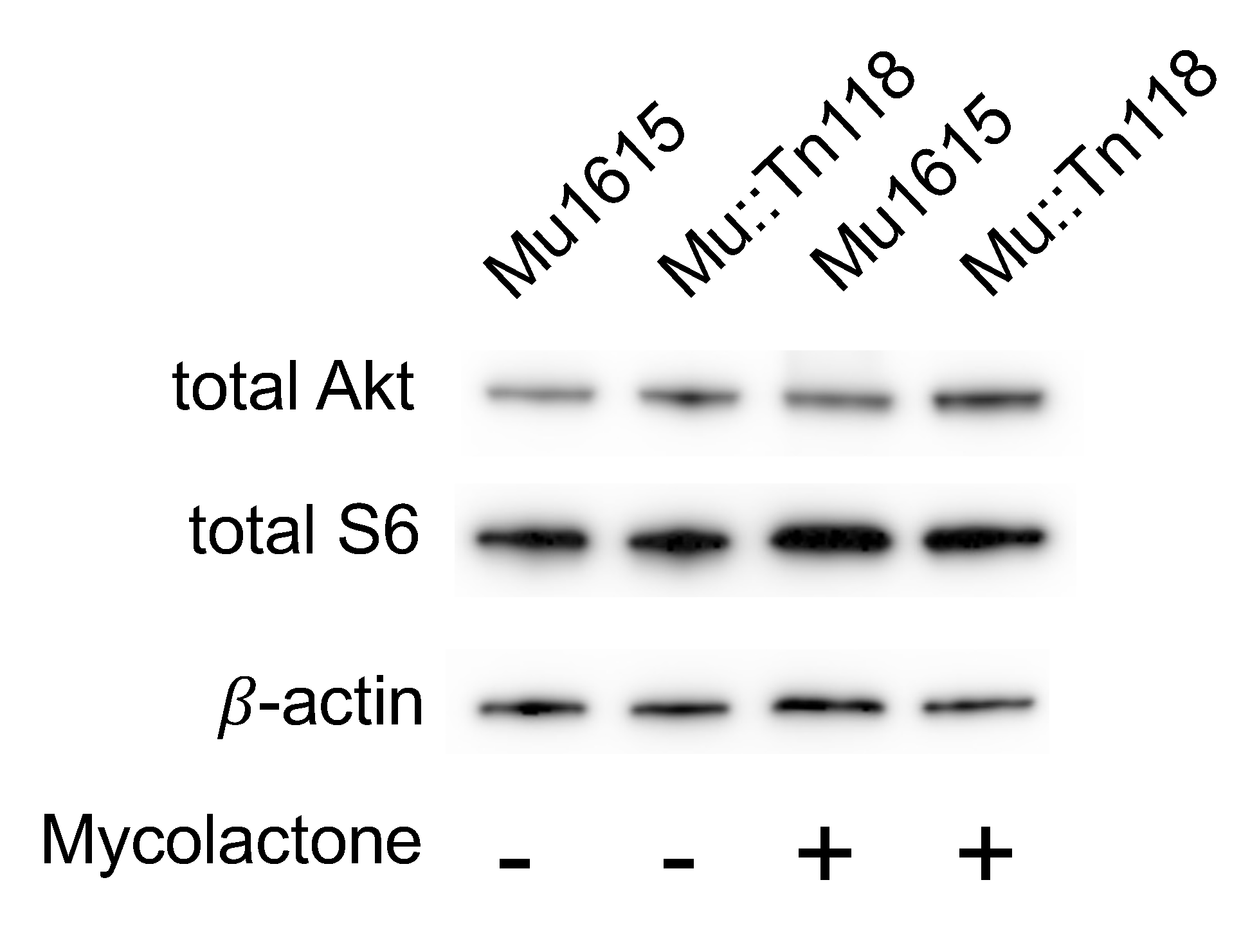

Supplement: Supplementary Figure 6 — Immunoblots were assayed from lysates of THP-1 monocyte-derived macrophages infected with Mu1615 or Mu::Tn118 at MOI 10 ± 100 nM mycolactone treatment, 72 hours post-infection for total Akt or S6. [file Image_6.tiff]
