# Supplementary material for: Induced Synthesis of Mycolactone Restores the Pathogenesis of Mycobacterium ulcerans In Vitro and In Vivo
Source: Front Immunol. 2022 Mar 24;13:750643. doi: 10.3389/fimmu.2022.750643 (PMC8988146; doi:10.3389/fimmu.2022.750643)
Supplement: Supplementary file 7 [file Table_1.pdf]

Supplementary Table 1: List of Antibodies

Antibody	Company	Catalog Number
LC3B (D11)	Cell Signalling Technology	3868
β-Actin	Cell Signalling Technology	4970
HA-Peroxidase	Sigma	12013819001
Rabbit IgG-HRP	Cell Signalling Technology	7074
p-S6 (S235/236)	Cell Signalling Technology	4857
p-Akt (S473)	Cell Signalling Technology	4060
HSP65	Invitrogen	MA1-7421
Mouse IgG-HRP	Cell Signalling Technology	7076
